# 

**Published:** 1894-01-13

**Authors:** 


### CONTENTS.

**A Medical Programme** - - - 231
**The Royal Hospital for Incurables** - - - 232
**On Modern Progress in Ophthalmic Medicine and Surgery.** By ROBERT BRUDENELL CARTER, F.R.C.S. - - 233
**Studies in Applied Sociology.** II.—The Making of American Doctors - - - 234
**Current Medical Topics.**—Operative Treatment of Tuberculous Joint Diseases - - - 235
**The Field of Science** - - - 236
**Annotations.**—Mince-pie Memoranda—Spoilt Patients—Medical Workers and Medical Thinkers - - - 237
**Notes and News** - - - 238
**Scraps and Gleanings** - - - 250
**Medical Progress and Hospital Clinics—**
Notes on Cases of Necrosis of the Lower Jaw in Children - - 239
Plastic or Fibrinous Bronchitis - - - 240
ROYAL ORTHOPÆDIC HOSPITAL—Wry-Neck - - - 242
NEW APPLIANCES AND THINGS MEDICAL.—The "Compactum" Midwifery Case - - - 242
MEDICAL PROGRESS—Pulmonary Tuberculosis - - - 243
Surgery of the Stomach - - - 244
NEW DRUGS AND PREPARATIONS—Agathin - - - 246
**The Institutional Workshop—**
HOSPITAL CONSTRUCTION.—The McLean Hospital, Waverley, Mass., U.S.A. - - - 247
**Medical Vacancies and Appointments** - - - 2[illegible]2

### EXTRA SUPPLEMENT.—THE NURSING MIRROR.

**News from the Nursing World.**—A Royal President—Trained to Prompt Action—Queen's Nurses—Poisoned Babies—Medical Women—Poor Pay—The Value of Labels—Patients before Probationers—Officially Recognised—Tea and Talk—Paris Asylum Attendants—Trained Nurses for Kandy—A Children's Hospital—The Alfred Hospital, Melbourne - - cxli
**On Nursing the Nervous and Insane.**—Introduction: The Nervous System - - - cxliii
**District Nursing.**—II. Preparation for District Nursing - cxliv
**Death in our Ranks—Royal Infirmary, Glasgow** - cxlv
**Nursing in the United States—Novelties for Nurses** - cxlvi
**Christmas Entertainments** - - - cxlvii
**Where to Go** - - - cxlviii
**The Muses' Looking Glass.**—An Englishman in France Ninety Years Ago - - - cxlix
**Minor Appointments—Appointments** - - - cxlix
**The Book World for Women and Nurses** - - - cl
**The Heroines Who Nurse Us?** - - - cl

